# Exploring the neurofunctional impairments and cognitive biases concerning food and body related stimuli in anorexia nervosa: An integrated EEG and eye-tracking study protocol

**DOI:** 10.1371/journal.pone.0299529

**Published:** 2024-03-28

**Authors:** Panagiotis Loizou, Georgia Panagiotou, Panos Zanos, Evangelos Paraskevopoulos

**Affiliations:** Department of Psychology, University of Cyprus, Nicosia, Cyprus; Istituto di Fisiologia Clinica Consiglio Nazionale delle Ricerche, ITALY

## Abstract

**Background:**

Patients with Anorexia Nervosa (AN) exhibit significant cognitive and neural disturbances compared to healthy individuals when processing food and body-related stimuli. These disturbances not only contribute to the manifestation and chronification of their pathological eating behaviour but also underscore the complex interplay of cognitive, emotional, and neurobiological factors in AN. However, the precise underlying cognitive and neural mechanisms of these disturbances remain a compelling area of investigation.

**Methods:**

This study presents a protocol developed for conducting a cross-sectional quasi-experimental study using a mixed model ANOVA approach with a crossover design. Our participants will consist of 20 patients with an active diagnosis of AN, 20 Overweight/obese individuals, and 20 Healthy Controls (HCs) with a normal BMI. An integrated eye-tracking and EEG methodology will be used in conjunction, with the primary aim of assessing participants’ cognitive and neural processing towards high and low-calorie food stimuli. On an exploratory level, by utilizing the same methods, the present study will also investigate AN patients’ responses towards high weight, normal weight, low weight, and self-body pictures, as well as towards images from the International Affective Picture System (IAPS) characterized by elevated valence and arousal levels. Additionally, behavioural methods such as yes or no questions, and self-reported questionnaires will be administered. The EEG and eye-tracking data will be analysed at early (50–300 ms) and late (350–500 ms) time intervals.

**Discussion:**

The investigation of the underlying cognitive and neural processes employed by patients with AN during the processing of food and body-related stimuli can help us develop a better understanding of the cognitive and neural mechanisms that contribute to the manifestation and maintenance of the disorder and assist in the development of more effective screening methods.

**Ethical approval and consent to participate:**

Ethical approval for the study has been obtained by the Cyprus National Bioethics Committee on 27.04.2023 (ΕΕΒΚ/ΕΠ/2023/19), and by the University of Cyprus (20.02.2023). Written informed consent will be obtained from all participants.

## Introduction

Anorexia Nervosa (AN) is a serious psychiatric disorder characterised by significant medical complications, disordered eating, weight loss, and body image distortion [1]. It has the highest fatality rate of all mental disorders [1,2], and is common amongst young Western women and adolescents below the age of 15 [3–5]. There are two AN subtypes: The Binge Eating/Purging subtype and the Restrictive subtype (AN-R) [6]. According to epidemiological findings, AN affects 0.9–4% of females and up to 0.3% of males in Europe and the United States [7–9].

Apart from health-related complications [10–13], patients with AN demonstrate substantial cognitive and neural impairments, particularly when processing food and body-related stimuli [14–18]. These disturbances contribute to the manifestation and chronification of the patients’ pathological eating behaviours [14,15,19–21]. However, the underlying mechanisms employed remain a topic of ongoing investigation as there are various interrelated factors (e.g., emotional, cognitive, and neurobiological) that contribute to the manifestation of the disorder [14,15,17,18,21].

### Emotion processing and regulation

Patients with AN exhibit difficulty in emotion processing and regulation, particularly in response to disorder-related stimuli (e.g., food and body-related stimuli) [22–24]. Exposure to such stimuli elicits heightened emotional responses in individuals with AN, indicating significant alterations in their emotion processing systems [22–24]. Research has shown increased activity in the right amygdala of patients with AN-R compared to Healthy Controls (HCs) when exposed to food pictures [25], as well as enhanced amygdala activity in response to body and shape stimuli [26,27]. Moreover, it was identified that patients with AN had reduced grey matter in the uncus, a key brain region for emotion processing [28]. The findings suggest heightened attentional responses to disorder-related cues [22] and alterations in brain regions implicated in reward processing [28,29]. These observations provide insights into disruptions in emotion processing and regulation areas among individuals with AN [28,29].

### Reward and punishment system dysfunctions in AN

Further research focusing particularly on the reward and punishment domain indicates that patients with AN exhibit altered functionality in the striatal circuitry, a key component of the brain’s reward system, demonstrating dysfunction of the dopaminergic circuitry and reward processing [30,31]. Based on resting state Functional Connectivity (FC), patients with AN also displayed lower connectivity compared to HCs in reward and habit-associated brain areas, such as the nucleus accumbens, superior frontal gyrus, ventral caudate, dorsal caudate, frontal and posterior regions [32]. More specifically, a decrease in the resting state FC between the nucleus accumbens and superior frontal gyrus was associated with greater symptom severity [32]. Patients with AN-R have also exhibited increased activation of the reward circuits in response to food stimuli as evidenced by a reward-conditioning task coupled with Functional Magnetic Resonance Imaging (fMRI) [33]. Furthermore, Magnetic Resonance Imaging and fMRI studies have also indicated an increased amygdala activation and maladaptive cingulate cortex activation, which are brain regions that are implicated in the processing of reward and punishment [29]. These dysfunctions have also been observed in recovered patients, suggesting potential irreversibility [29].

Additionally, it was shown that patients with AN also exhibited higher punishment sensitivity, which is believed to be explanatory of the patients’ rigid cognitive styles, perfectionism, and harm-avoidant behaviours [34]. These disruptions in the reward and punishment networks of AN patients might also account for their maladaptive behaviours such as the fear of weight gain and resistance to food consumption [34]. Moreover, it was observed that the severity of symptoms in individuals with AN was linked to their punishment and reward sensitivity [34], suggesting that these factors could potentially serve as risk factors for developing AN [34].

### Subjective ratings of food and body stimuli in AN

In accordance with prior findings, on an explicit level, patients with AN demonstrate diminished subjective interest in food, reduced craving, and negative evaluations for food pictures compared to HCs [35–39]. More specifically, they rate High Calorie (HC) food pictures as less palatable and display negative valence ratings for food [35,40]. Similarly, when it comes to body attractiveness, they rate underweight bodies as more attractive and display lower ratings for normal and overweight bodies [41–44], they also exhibit implicit and explicit biases against fatness [45]. These findings emphasize that the distorted perceptions observed in individuals with AN extend beyond favouring thin and emaciated body types, as they also have negative attitudes towards both normal and overweight body types [9,16].

### Attentional bias towards food and body stimuli

Attentional biases play a crucial role in understanding the abnormal processing of food and body stimuli in AN [18,21,46,47]. Patients with AN demonstrate unconscious prioritization of food stimuli [15] causing an increased distraction as shown in working memory tasks [15,47]. Patients with AN were also significantly distracted by food words with HC content, while words with Low Calorie (LC) content had little to no effect [15]. Reflecting on the importance of attentional biases, prior studies reported a positive correlation between patients’ severity and their attentional bias towards food cues [20,48], also, supported that differences in attentional biases towards food stimuli indicate differences in food craving predispositions [49], as well as differences in the reward system functioning [50]. Moreover, AN patients’ unconscious prioritization of food and body-related stimuli was also found to be linked with negative emotions and high concerns [35,47,51–56]. Thus, AN patients’ attentional bias towards food stimuli might also signal a threat-related response. However, it is still unclear whether AN patients’ attentional biases towards food pictures indicate:

- High craving and hedonic motivation [11,49], which might also be due to the existence of an adaptive mechanism that gets activated in a state of undernourishment [35].
- High worry and concerns about food intake, due to increased salience that activates a threat response that might be associated with further dietary restriction [49,53,57].
- Both high craving and hedonic motivation as well as high worry and concerns [49].

As highlighted in prior studies, it is also important to underline that the mechanisms employed during late time-windows (top-down processing) (> 300 ms) that are showcased by the maintenance of attention towards the target stimuli differ from those utilized during early time-windows (bottom-up processing) (< 300 ms) [56,58]. Therefore, methods that provide excellent temporal resolution and time-sensitivity should be utilised to further elucidate the mechanisms employed by AN patients during the processing of food and body-related stimuli. These time-sensitive methodologies will allow the distinction between early and late processing and the focus on time-related information [59].

### Early processing

Early attentional processing reflects bottom-up attentional processing and corresponds to participants’ early sensory processing and selection procedures [59]. Research evidence showed that patients with AN exhibit early attentional bias [40] and neural responses (Early Posterior Negativity; EPN) [35,53] towards disorder-related stimuli.

According to Electroencephalography (EEG) findings obtained from a systematic review, patients with AN displayed higher EPN than HCs when presented with HC and LC food pictures compared to neutral and emotional pictures (200–300 ms) [59]. Similarly, a study that used a rapid serial picture presentation identified that during early time intervals (< 300 ms) patients with AN had increased neural responses (e.g., EPN) towards both HC and LC food pictures, while HCs exhibited early attentional bias only in HC food pictures which was attributed to high levels of craving [58]. This finding suggests that individuals with AN have a persistent preoccupation with food, potentially driven by the implicit aim of restricting their exposure to these stimuli [58].

In accordance with the aforementioned findings, participants with AN demonstrated heightened neural activity during early processing regardless of the caloric value of the presented food stimuli [53]. During the early automatic processing stage (< 300 ms), a study that used Magnetoencephalography examined the neural response of adolescent patients with AN towards HC and LC food stimuli; and revealed a significantly high neural response towards food cues, providing evidence for abnormal early motivational processing [35]. With regards to recovered patients, it was shown that they also exhibited attentional biases at a slightly later latency, which might be considered as a sign of recovery [53]. Additionally, patients’ attentional biases were positively correlated with the severity of their pathology [47,48].

Similar findings were also observed in response to body-related stimuli, where patients with AN showed an early vigilance towards unattractive bodies or body-parts followed by a subsequent decrease of attention (vigilance-avoidance theory) [60]. Similarly, patients with AN focused longer on their own subjectively unattractive body parts as well as on the unattractive body parts of obese body-stimuli with an even greater attentional bias compared to HCs [43,54].

Contrary to the prevailing research evidence, there are studies that yielded conflicting findings. For instance, an eye-tracking study that explored AN patients’ early attentional bias towards food stimuli did not identify any significant findings [36]. Similarly, a study that employed a behavioural approach-avoidance paradigm found no biased tendencies towards food and body stimuli in patients with AN [61]. These findings highlight the urgency to further explore the underlying mechanisms to better understand the cause of these discrepancies.

### Late processing

Apart from early attentional bias, patients with AN also demonstrated a later avoidance (diminished attention maintenance) as shown in a recent eye-tracking study [56]. The examination of later attentional processing can provide insights into diminished attention maintenance (> 300 ms) [56]. Later attentional processing is characterised by heightened Late Positive Potential (LPP) [59]. LPP is also linked to top-down processing and reflects the participants’ subjective preferences and motivational significance to food related stimuli [59]. Thus, individuals’ preferences and motivations have significant implications for understanding the underlying mechanisms involved in the processing of food and body-related cues as they affect later attentional processing.

By employing an EEG methodology, empirical evidence showed that adolescent patients with AN exhibited heightened frontal activity during a subsequent regulatory response (350–500 ms) towards food cues [35]. This regulatory response effectively diminished the patients’ initial neural response towards food cues indicating the existence of a regulatory mechanism responsible for the suppression of AN patients’ desire to eat [35]. Additionally, in eye-tracking studies [36,53], while both HCs and AN patients had longer fixation times on food compared to non-food pictures, the HCs exhibited significantly longer fixation time towards food pictures compared to patients with AN, once again revealing the regulatory attempt of AN patients to suppress their exposure to food stimuli. Similar findings were also reported for body-related stimuli using an EEG approach [62], whereas adolescent patients with AN exhibited greater cognitive processing when viewing pictures of underweight compared to normal-weight and overweight women during later time windows (850–1250 ms), while HCs displayed higher amplitudes in response to normal-weight individuals during later time windows [62].

Contrary to previous findings, by using an eye-tracking and Event Related Potentials methodology during single stimuli presentation, it was observed that patients with AN exhibited increased fixation time towards HC food stimuli compared to LC food [40]. However, in the presence of competing HC and LC food pictures, patients with AN initially showed short-term attentional orientation towards HC pictures, followed by a bias away from HC pictures and/or towards LC food pictures [40]. In the same study, the participants with AN rated HC food pictures as more negative than LC food compared to HCs [40]. These findings show that HC pictures were potentially perceived as aversive and threatening by patients with AN, leading to heightened concerns and attentional bias [40]. Also, these findings suggest potential differentiations in the processing mechanisms employed when stimuli are presented with or without distractors [40]. Furthermore, these findings might also indicate the existence of distinct cognitive regulation processes during later processing stages [58]. For instance, in line with the observed early vigilance and later avoidance mechanism reported in previous studies [48,62,63], patients with AN demonstrated a tendency to direct their attention away from threatening HC food stimuli during the later processing of competing food stimuli [40]. This attentional pattern is similar to the one seen in individuals with social anxiety disorder, where initially they allocate more attention towards the threat stimuli but later as a coping mechanism they disengage and avoid them [64]. An alternative interpretation of the findings suggests that patients with AN might exhibit later avoidance of HC stimuli due to heightened craving for LC food stimuli [40], which aligns with the high craving and hedonic motivation theory [11,49].

Nonetheless, in contradiction to the high craving and hedonic motivation theory, EEG and Event Related Potential findings during a food recognition test that used both semantic and pictorial food stimuli showed that patients with AN regardless of their hunger state had a reduced activation during later processing (400–800 ms) compared to HCs [65]. This finding indicates that patients with AN have an impaired allocation of their attentional resources towards food stimuli, which is reflected by their reduced hedonic motivation towards food consumption [49,65]. In contrast, the later processing of HCs towards food stimuli was positively associated with their internal state of hunger, as they exhibited enhanced amplitudes for food-related pictorial stimuli when they were in a hungry state [65]. This indicates a differentiation in how hunger affects the processing of food-related stimuli between the two groups.

### Study rationale and methodological innovation

Taken together, the aforementioned findings have shown that patients with AN exhibit altered neural and cognitive processing towards food related stimuli [65]. The findings have also highlighted the complex nature of AN patients’ responses towards food and body-related stimuli, which are influenced by underlying mechanisms affected by various factors such as maladaptive neurophysiological processing, motivational significance of the presented stimuli, subjective ratings, craving, hunger state, and contextual factors such as the presence of competing stimuli. Thus, a comprehensive understanding of the underlying mechanisms employed remains elusive. Moreover, the limited localization of findings in prior studies can be attributed to methodological approaches that lack temporal resolution (e.g., fMRI) which restricts the differentiation between both early and later effects as well as the use of distinct early and later mechanisms. Hence, the innovative methodology we propose aims to exploit a triangulation of research methodologies including well-established neurophysiological methods [66] combined with the use of eye-tracking and complemented by self-reporting questionnaires and behavioural (yes/no questions) questions. This multifaceted approach with high temporal resolution and time specificity will enable us to disentangle the contribution of different cortical regions for each of these mechanisms, which will provide us with a comprehensive understanding of the phenomena under investigation.

## Current study

The present study aims to investigate AN patients’ neural and cognitive processing of food-related stimuli (HC and LC food pictures) by using a multimodal integration of electrophysiological (e.g., EEG, and eye-tracking), behavioural (e.g., yes/no questions), and self-reported methods (e.g., questionnaires). By combining these techniques, we aim to capture the complex interplay between temporal dynamics, brain activity, FC, and attentional biases of AN patients during the processing of food stimuli, which will assist in the comprehensive understanding of the cognitive and neural mechanisms employed. Additionally, by using the same measurements, exploratory analyses will also be performed in response to:

1. High weight (HW), Normal Weight (NW), Low Weight (LW), and self-body stimuli.
2. Pictures from the International Affective Picture System (IAPS).

Our main hypotheses regarding the cognitive processing of paired food stimuli are:

1. Patients with AN will exhibit a higher early attentional bias (50-300ms) towards food stimuli (particularly the HC food stimuli). This initial attentional bias will be followed by a subsequent avoidance response (350-500ms), indicating a shift away from the HC food stimuli.
2. The total gaze duration of patients with AN towards HC food pictures is expected to be less than the total fixation time of the HCs and obese/overweight participants.
3. The total gaze duration of patients with AN towards HC food pictures will be positively associated with their responses to the behavioural questions assessing palatability and craving.
4. The total gaze duration of patients with AN towards HC food pictures will be positively associated with their responses in the self-report questionnaires that measure symptoms and concerns of eating disorders (Eating Attitudes Test-26; EAT-26) and body-image acceptance (Body Image-Acceptance and Action Questionnaire; BI-AAQ).
  Our main hypotheses regarding the neural processing of food-related stimuli based on whole-head analysis are:
5. Patients with AN will exhibit heightened EPN during the early processing stages (50–300 ms) of food stimuli compared to HCs and Overweight/obese participants, especially for HC food stimuli. A relative negative deflection in temporo-occipital brain areas (reflected in posterior EEG electrodes) is expected.
6. Compared to HCs, patients with AN are expected to exhibit increased brain activation in the prefrontal cortex and demonstrate statistically significant differences in the posterior regions.
  Our hypothesis concerning FC is:
7. Patients with AN will demonstrate substantial FC differences compared to HCs. These FC differences are particularly expected in regions that involve emotional, reward, and visual processing (e.g., corticolimbic circuit, visual and auditory networks, the default mode network, and visuospatial and somatosensory networks).
  Our hypothesis regarding the participants’ behavioural responses is:
8. Patients with AN are expected to exhibit lower overall scores for palatability and craving compared to HCs and Overweight/obese individuals. Specifically, patients with AN are expected to rate LC food as more palatable than HC food and have higher cravings for LC food compared to HC food.

## Materials and methods

### Research design

A cross-sectional quasi-experimental design will be employed using a convenience sample approach. The participants will be assigned to their respective groups based on their diagnosis or characteristics. A mixed model Analysis of Variance (ANOVA) approach with a crossover design will be employed. The factors in the model will include the category of stimuli and the participants’ group. An integrated eye-tracking and EEG methodology will be employed to assess AN patients’ cognitive (e.g., attention deployment) and neural processing (e.g., neural activation, and FC) (dependent variables) in response to the presented visual food stimuli (independent variables). For the main analyses, the visual stimuli will consist of food pictures (e.g., single and competing HC and LC pictures). On an exploratory level, the present study will also investigate AN patients’ processing towards body-related pictures of different weight status, and IAPS pictures (e.g., high on valence, and high on arousal).

### Participants and recruitment procedure

Based on the results of an a priori power calculation performed using G*Power version 3.1.9.7, to ascertain the requisite statistical power for our study, we have determined that a minimum of 21 participants are required. The calculation was performed for an F-test, with an effect size *f* = 0.43, a significance criterion of α = 0.05, and a desired power of 0.95. The effect size was chosen based on the ’’category’’ × ’’participant group’’ interaction effect reported in a similar study (η_p_^2^ = 0.16) [67]. For the purpose of power-calculation, the number of groups (between-subject factors) was set to three (HCs, AN patients, and Overweight/obese participants), and the number of measurements (within-subjects factors) was also set to three (HC pictures, LC pictures, and Random objects). Thus, by using a 3x3 analysis, G-Power estimated an actual power of 0.956, which is above our threshold that was a-priori set to 0.95. According to the results, each of our groups should consist of a minimum of 7 participants. In consideration of the sample-size calculation results and to accommodate potential variations in statistical power arising from specific protocol details that distinguish our study from others [67], we aim to recruit a total of 60 participants. These participants will be equally divided into three groups: 20 adults diagnosed with AN (Restrictive or Binge Eating/Purging subtype) that will be recruited from public hospitals, treatment centres, and through invitations distributed via social media and emails sent by the University of Cyprus Department of Psychology; as well as 20 HCs and 20 Overweight/obese volunteers that will constitute the control group. Control group participants will be recruited through social media and email invitations and will be matched to AN patients based on gender and age.

With regards to participants’ dropout rate, as the participants will be subjected to the complete experimental procedure within the period of a single lab visit lasting around two hours and no intervention exists in our protocol, we do not expect a high drop-out rate. Nonetheless, in the unlikely case that a participant withdraws before completing the set of measurements, his/her data will be excluded from the analysis and the number and rate of such dropouts per participant group will be reported in the following research report.

### Inclusion and exclusion criteria

All the participants should have a good comprehension of the Greek language to participate in the study. For the experimental group, participants need to have a BMI equal to or lower than 18,5 and an active diagnosis of AN. Additionally, the participants with AN need to score above 20 on the Eating Attitudes Test-26 (EAT-26) questionnaire. Regarding the control groups, HCs and Overweight/obese participants are expected to have a BMI within the ranges of 18.5–25 and over 25 respectively. Furthermore, for the HCs and Overweight/obese individuals, their scores on the Depression Anxiety Stress Scale questionnaire (DASS-21) should fall within the moderate range for stress (0–14), depression (0–9), and anxiety symptoms (0–7). Also, the control group participants (Healthy individuals and Overweight/obese individuals), will undergo screening for eating disorders such as AN, Bulimia Nervosa, and Binge Eating Disorder. This screening will involve the administration of the EAT-26 questionnaire alongside questions included in the self-report questionnaire that will be administered such as: “Do you have a history of eating disorders?”, “Do you believe that you have experienced an eating disorder such as AN, Bulimia Nervosa, or Binge Eating Disorder, in the last 6 months?”, “Have you ever seen a doctor or a mental health professional for an eating disorder?”. Participants disclosing a current or past history of eating disorders, as indicated by any positive responses to the self-report questions or an EAT-26 questionnaire score exceeding the cutoff of 20, will be deemed ineligible for inclusion in the study. The control group participants will be matched to the experimental group in terms of gender and age. Additionally, following their replies in the demographic questionnaire, participants with colour blindness, intellectual disabilities, neurological disabilities, alcohol or drug abuse, psychotic disorders, pervasive developmental disorders, or visual impairments will be excluded from the study. Lastly, to ensure the clarity and integrity of our research, we have also chosen to exclude individuals with a history of bariatric surgery. This is based on evidence indicating that bariatric surgery induces changes in how individuals perceive and assess body shapes and weight [68]. Such effects have the potential to influence the processing of body-related stimuli presented in the current study.

### Procedure

Prospective participants will be contacted by the main researcher to schedule an appointment and address any inquiries. Participants will also be instructed to consume soft food, and avoid smoking, eating, and drinking alcohol or caffeine-containing beverages three hours before the experiment. On the scheduled appointment at the University of Cyprus, participants will be asked to complete a consent form indicating their voluntary participation and a demographic questionnaire that will assess their eligibility to participate. Then, they will be requested to fill out the DASS-21, EAT-26, and BI-AAQ questionnaires using Google Forms.

Eligible participants will be seated comfortably at a distance of 60 cm from a computer monitor. The eye movement recording will be calibrated, and the participants will wear the portable eye-tracking device concurrently with the EEG electrode cap, which will be placed to ensure precise electrode positioning and facilitate the integration of EEG and eye-tracking methods. The EEG and eye-tracking measurements will be used simultaneously during visual stimulation paradigms (six blocks in total): three with food-related stimuli, two with body-related stimuli, and one with IAPS pictures. Each block will last around five minutes, and the entire experiment will take about 90 minutes. Throughout the procedure, participants’ movement will be relatively restricted.

There will be a total of three food-related blocks. The first food-related block will consist of 60 single food-picture trials (30 HC and 30 LC pictures) that will be randomly presented on a white plate and followed by a palatability question. The second block will consist of 30 paired food-picture trials, followed by a craving-related question about which food they would prefer to eat. In the third block, the same food stimuli will be presented alongside random objects on a grey background without any subsequent behavioural questions.

With regard to the body-related blocks, a total of two will be present. The first block will include 30 pictures of LW bodies, 30 pictures of HW bodies, 30 pictures of NW bodies, and 30 self-body pictures with the participants’ faces covered for anonymity. Each picture will be followed by a question about its attractiveness. The second block will consist of 60 paired body-picture trials featuring LW and HW body pictures without any subsequent behavioural questions.

Lastly, the IAPS block will consist of two sets of single pictures (the high valence-low arousal and the low arousal-high valence pictures) which will be presented randomly without any subsequent behavioural questions.

**Fig 1:**
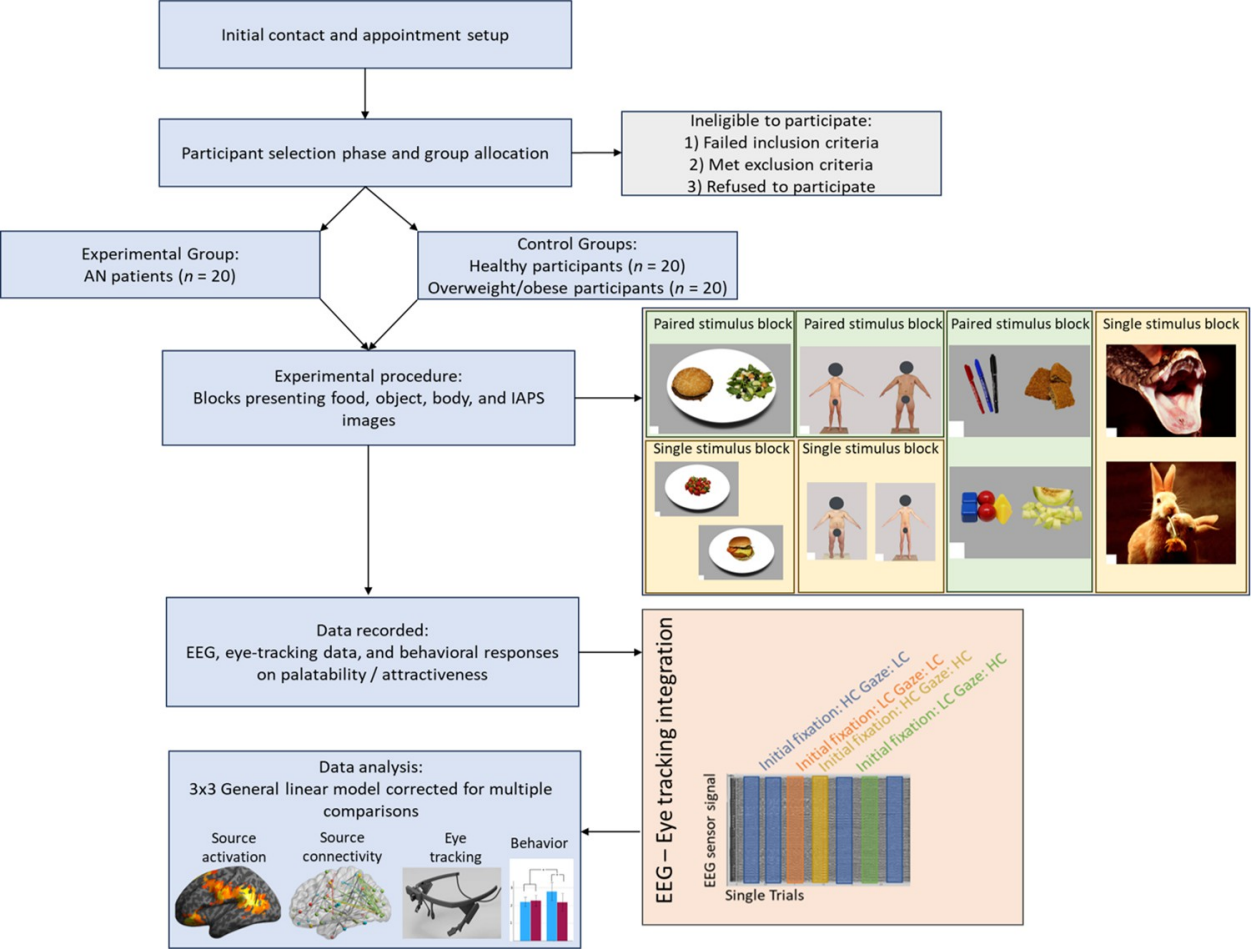
Study protocol flowchart.

Fig 1 provides a visual overview of the study protocol, depicting the sequential journey from initial contact and appointment scheduling to participant selection, group allocation, EEG and eye-tracking integration, and the presentation of food, body, and IAPS blocks. This figure offers a concise guide to the study’s execution.

### Measurements

#### Physiological measures

A Biosemi *EEG* system with 128 active electrodes will be used to capture participants’ neural responses to the presented stimuli. It will record, localize, and characterize neural activity, FC, and whole-brain activations. The EEG data will be recorded and analysed during an early time window (50–300 ms), which reflects early attentional bias, and a later time window (350–500 ms) indicating later avoidance.

Concurrently, a state-of-the-art wearable eye-tracker headset (PupilLabs Core) will be used to monitor participants’ eye gaze and eye fixations. This will enable the assessment of their attentional processing during both early (50–300 ms) and later (350–500 ms) stages in response to the presented stimuli.

#### Behavioural measures

Following food and body-related trials, participants will also be requested to respond with a button press to yes or no questions. Their answers will indicate their subjective ratings regarding food palatability and craving (e.g., ’’Was the presented food tasty?’’ and ’’Which food would you prefer to eat?’’), as well as their subjective attractiveness ratings for the presented body-related stimuli (e.g., "Is the presented body attractive?" and "Which is the most attractive body?").

#### Self-report questionnaires

A short Likert-type demographic survey will assess participants’ background information, including age, sex, race, ethnicity, education, marital status, employment, height, weight, health status (e.g., underlying health conditions, developmental disorders, active diagnoses, mental disorders, drug dependency, visual impairments), physical activity, medication use, current hunger level, and eating preferences.

DASS-21, which is a 21-item self-administered Likert-type questionnaire will be used to assess depression, anxiety, and stress symptoms [69]. DASS-21 has three subscales (Depression, Anxiety, and Stress) with seven items each [69] that range from 0 ’’Did not apply to me at all–Never’’ to 3 ’’Applied to me very much’’, or ’’Most of the time–Almost always’’. The Greek version of the DASS-21 has shown satisfactory reliability (α = 0.85, 0.84, and 0.84) and validity, including convergent and discriminant validity [70].

To assess participants’ behaviours and attitudes related to disturbed eating and body weight, the Eating Attitudes Test-26 (EAT-26) will be used. EAT-26 is a widely used self-report questionnaire that consists of 26 questions organized into three subscales: diet (13 questions), Bulimia and food preoccupation (6 questions), and oral control (7 questions) [71,72]. Responses are rated on a Likert scale ranging from always (3) to never (0) [71,72]. A total score of 20 or higher indicates abnormal eating behaviour and possible eating disorders [72]. The EAT-26 has been adapted for the Greek population, demonstrating good psychometric properties [73–75], including acceptable discriminant and criterion validity [75] and internal consistency [75]. Cronbach’s alpha values for the total EAT-26, dieting, bulimia, and oral control subscales were found to be .80, .79, .65, and .64, respectively [75]. In summary, the EAT-26 is a well-established self-report questionnaire for assessing disturbed eating behaviours and attitudes related to body weight. Its adaptation for the Greek population has demonstrated robust psychometric properties, making it a valuable tool for studying eating disorders [75,76].

BI-AAQ will be utilized to assess our participants’ cognitive flexibility and body image acceptance. BI-AAQ is a 12-item questionnaire that uses a seven-point Likert scale where higher scores indicate greater inflexibility [77]. The BI-AAQ has demonstrated excellent reliability (α = .92) and convergent validity with measures of awareness, acceptance, body shape, and disordered eating habits [77]. The Greek translation of the questionnaire, validated with Greek and Cypriot undergraduate students (*n* = 240), showed unifactorial structure, good internal consistency (α = 0.95), and satisfactory item-total correlations (except for item 6) [78]. These findings confirm the validity and reliability of the Greek version of the BI-AAQ [78].

#### Primary and secondary outcomes

The primary outcome of this experiment is to assess the significance of the Group × Condition interaction in the statistical analysis of EEG source activation and cortical connectivity data during both early and later time windows, revealing differences in neural and cognitive processing mechanisms between HCs and AN patients. Specifically, a *p*-value < 0.05 corrected for multiple comparison via Family-Wise Error (FWE) correction in any cortical region activated, will indicate significant differences in the cortical activation pattern of the three groups in processing LC food pictures, HC food pictures, or random objects.

The secondary outcome of this experiment is to assess the significance of the interaction between Group and Condition in the statistical analysis of the eye-tracking data for early and later processing time windows. Specifically, a *p*-value < 0.05, corrected for multiple comparisons using the Bonferroni method, for the duration of gaze engagement with each stimulus, will indicate significant differences in the patterns of gaze engagement among the three groups during the processing of HC food stimuli, LC food stimuli, and random objects.

Another secondary outcome of this experiment is to assess the significance of the interaction between Group × Initial Response × Gaze Duration in the statistical analysis of the combined EEG and eye-tracking data. Specifically, a *p*-value < 0.05 corrected for multiple comparison via FWE in the triple interaction, will indicate significant differences in the cortical activation pattern of the three groups when avoiding the processing of a food stimulus.

### Data analyses

#### Self-reporting questionnaires and behavioural data

A mixed-model ANOVA with a crossover design will be employed, considering both within-subject and between-subject factors for both self-reporting questionnaires and behavioural data. The statistical analyses will be conducted using the IBM-SPSS version 27. Descriptive and inferential statistical analyses will be employed, with a significance threshold set at *p* < 0.05. The primary aim of these analyses is to explore the magnitude and direction of the potential associations between the variables. Post-hoc analyses will be conducted if required to further explore significant results.

#### EEG analyses

The EEG pre-processing will be conducted using software Brain Electrical Source Analysis software (BESA research, version 7.1, Megis Software, Heidelberg, Germany). Initially, the data will be visually inspected, and bad channels will be interpolated, while artifacts generated by blinks or eye movements will be corrected using an adaptive correction method. Continuous data will be separated into epochs of 1000 ms, including 200 ms of a prestimulus interval. Epochs will be baseline corrected using the interval from −100 to 0 ms. Data will be filtered offline with a high-pass forward filter of 2 Hz, a low-pass zero phase of 30 Hz, and an additional notch filter at 50 Hz. The averages will be calculated separately for the 3 conditions (HC pictures, LC pictures, random objects).

Following the pre-processing using BESA research software, the Global field power will be extracted from the average responses of all trials. Sensor space data will undergo non-parametric analysis to compare between conditions, identifying time-windows with significant activity differences. Concerning the source activity estimation, Current Density Reconstructions (CDRs) will be calculated on the neural responses of each stimulus category (HC pictures, LC pictures, random objects) and for each subject. A realistic approximation of a standardized finite element model template will be created from an averaged head using 50 individual magnetic resonance imagings in Talairach space that will be used as a head model for the CDRs. The images will be then smoothed through an isotropic Gaussian kernel of 7 mm full-width at half-maximum as provided by BESA. CDRs will be calculated using Low-Resolution Brain Electromagnetic Tomography for the source’s activity estimation in the significant time-windows to solve the inverse problem using an average Finite Element Model for the forward. The CDRs will be estimated for two pre-defined time windows corresponding to early (50–300 ms) and late (350–500 ms) processing stages. CDRs will co-register into the Montreal Neurological Institute space using Statistical Parametric Mapping 12. For each time window (early and late) the following analysis will be performed on the corresponding CDRs.

Data will be exported in nifti format and significant differences in cortical activity strength between conditions/groups (Healthy participants, Overweight/obese participants, AN patients) and within-subjects factors Conditions (LC pictures, HC pictures, random objects) will be identified modelling the above-mentioned design via the Sandwich Estimator Toolbox for Longitudinal & Repeated Measures Data [79] both running on Matlab (Math Works Inc., Natick, MA, USA). Specifically, a 3 × 3 mixed model analysis will be implemented, with between-subjects factor Group (AN patients, Healthy participants, Overweight/obese participants) and within-subjects factors Condition (LC pictures, HC pictures, and random objects). The predefined contrasts estimated via this analysis will evaluate: 1) the main effect of Condition (whether the cortical sources activated in the processing of LC food pictures, HC food pictures, and random objects differ) independently for each group, 2) the main effect of Group independently of the Conditions [whether the cortical sources activated in the processing in each group as a response to food stimuli differ, independently of the type of food (i.e. LC, HC food pictures or random objects)], 3) the group differences in the LC Vs. HC food processing (reflected in the double-sided contrasts of the 3 × 3 interaction of the factor Condition and the factor Group). Wild bootstrap will be used to estimate nonparametric inferences for the defined contrasts using 10000 permutations, while the threshold-free cluster enhancement approach will be used to calculate FWE corrected *p*-values at a voxel-cluster level, effectively controlling for multiple comparisons independently for each contrast. The reporting threshold will be *p* < 0.05 corrected for multiple comparison. This method ensures proper control of FWE for each contrast while simultaneously provides better sensitivity than other methods over a wide range of test signal shapes and signal-to-noise-ratio values. Then, the time-course of the activity of each voxel will be extracted and time-delayed. Mutual Information will be used to estimate directed connectivity graphs reflecting the cortical network of each condition and each participant. These graphs will be subjected to statistical analysis, applying again the above-mentioned design via the Network Based Statistics toolbox [80], following a False Discovery Rate correction at the level of *p* < 0.05 to identify significant cortical network differences between conditions/groups, as previously performed by the authors [66].

Following previously published procedures [81], the FC of brain networks underlying the processing of food and body related pictures will be evaluated using the MATLAB toolbox Hermes to calculate the adjacency matrix from the voxel time-series. The toolbox Network Based Statistic will be used to statistically identify significant connections in the graphs, using a General Linear Model approach. For the statistical analysis of the data, a 3 × 3 mixed model ANOVA design will be followed, with within-subjects factors for each category of stimuli. The significance level will be set to *p* < 0.05 corrected for multiple comparisons via FWE correction.

#### Eye-tracking analyses

Concerning the pre-processing, a calibration procedure will be followed to ensure optimal gaze data quality, and then a Region Of Interest (ROI) analysis will be performed to estimate the duration of gaze engagement with each stimulus condition. Both for the single and paired food blocks the area occupied by the food items will be marked as the ROI. Each category of stimulus will be presented within an eye-tracking ROI specific to the condition, and the participant’s gaze duration within the ROI of each image in the respective condition will be measured via the eye-tracker. The same approach will be followed in the body-related blocks and IAPS block as part of the exploratory analyses.

Eye movements during all blocks will be registered binocularly at a resolution of 720p and a refresh rate of 60 Hz. The recorded data will include fixation time and gaze duration for each ROI. A fixation will be counted if the participants’ gaze was directed to the predefined ROIs for at least 100 ms. The total fixation time within each ROI will be calculated and corrected for the size of the food item (ROI area/area of the whole picture).

Regarding gaze duration, data will be subjected to a statistical analysis following the design reported for the EEG data by utilizing SPSS for statistical analyses. Specifically, a 3 × 3 mixed model design will be estimated with between-subjects factor group and within-subjects factor condition. The pre-defined contrasts will remain identical to the EEG data analysis ones. A Bonferroni multiple comparison will be applied via SPSS, and the significance threshold will be set at *p* < 0.05.

The normality of the distribution of the analysed variables will be assessed through normality testing and manual visual inspection of charts, and the group differences in gaze data will be ascertained using ANOVA. An α-level of .05 will be set to determine statistical significance.

#### Integrating EEG and eye-tracking

In the paired food blocks (e.g., HC Vs. LC picture), each trial will be categorized as one or the other condition on the basis of the gaze duration of the participant in each stimulus, along with the information on which stimulus the participant first focused on. In more detail, if a participant initially fixates on the image of the LC picture food but has a longer engagement with the HC picture, this trial will be categorized as a LC trial. If the participant fixates on the LC image and has a longer engagement with this, then this will be a LC trial. Correspondingly, if the participant initially fixates on the HC food image and engages for a longer duration to the LC one, this will be a HC trial, etc. Via this approach, the EEG data will compare initial response and engagement Vs initial response and avoidance for each data condition. The CDRs corresponding to this categorization of the trials will be subjected to a statistical analysis using a 3 × 2 × 2 mixed model design. This design will include the between-subjects factor group (HCs, AN patients, and Overweight/obese participants) and the within-subjects factors initial response (HC food pictures, LC food pictures) and gaze duration. The same method will be followed for the categorisation of the paired blocks that comprise of a food image (HC or LC picture) Vs. a Random object.

#### Data management

The data collection process and data management will be conducted by the PhD student Panagiotis Loizou under the supervision of Dr. Evangelos Paraskevopoulos. All the collected data will be used exclusively for the purpose of the present study and will be kept electronically on a password protected laptop that will not have access to the Internet and will be accessible only by the researcher (PL) and supervisor (EP) of the study. Following the GDPR guidelines, as well as anonymization and pseudonymization processes, the collected data will be converted into codes by the researcher so that individuals cannot be identified in order to ensure that after the data collection phase none of the data used will relate to an identifiable natural person.

The code of each participant will be used by the researcher (PL) and his supervisor (EP) in case of withdrawal from the study in order to track and permanently delete the participant’s data.

Also, before participating in the study, all potential participants will be informed about their right to withdraw at any stage during or after their participation without the need to explain their decision.

#### Trial status

The initiation of the recruitment phase is scheduled to begin around May 2024, and will continue until the intended sample size is achieved. Only the principal investigator will have authorization to access information that could reveal the identity of individual participants.

## Discussion

By utilizing an integrated eye-tracking and EEG methodology in conjunction with behavioural and self-report approaches the present study aims to investigate the neural and cognitive processing towards food related stimuli in patients with AN. By following the same approach and on an exploratory level, the neural and cognitive processing of AN patients towards body-related stimuli of varying weight status and IAPS pictures will also be investigated. The uniqueness of the study lies in its comprehensive methodological approach, which will allow for a thorough assessment of the implicit and explicit mechanisms employed by patients with AN as well as its inclusivity of disorder-related stimuli (e.g., body and food stimuli). Another key strength of the study is the inclusion of a control group that will consist solely of Overweight/obese participants. This is because the examination of the neural and cognitive procedures across a wide range of weight-related categories (overweight or obese participants, HCs, and individuals with AN) will provide us with insights about the neural and cognitive mechanisms that contribute to disordered eating and weight-related issues. Additionally, following the study design, time-specific analysis that distinguish between early and late time windows will be employed. Furthermore, the study will include the presentation of both single and paired stimuli which will allow the examination of how the cognitive and neural processes unfold over time. Apart from food images, the exploratory investigation of IAPS pictures will also provide additional evidence regarding the involvement of emotional processing systems in patients with AN, revealing insights into their role in the processing of stimuli related to the disorder. Lastly, the assessment of AN patients’ FC in response to both food and body-related pictures is a novel contribution to the whole research field as no prior studies, to the best of our knowledge, have investigated this important aspect.

In conclusion, the present study protocol outlines a rigorous approach for the investigation of the cognitive and neural processing of food and body-related stimuli in patients with AN. By doing so, we aim to advance the understanding of AN, to improve the prognostic and treatment approaches, and ultimately to benefit patients with AN and the society as a whole. The present study also holds the potential to significantly impact the health and societal sectors, particularly towards the development of more effective screening methods aimed to mitigate the financial burden associated with AN treatment [82].

The results of the study will be made available in a variety of ways. Firstly, research reports that provide a concise overview of the study’s findings and their policy and decision-making implications will be created and forwarded to policymakers, and governmental organizations. Additionally, public events such as talks, lectures, and webinars will be planned to convey and promote the interpretation of our research findings. Lastly, research articles will be produced for publication in peer-reviewed journals and presentations at international conferences.
